# Host Genetics of Cytomegalovirus Pathogenesis

**DOI:** 10.3389/fgene.2019.00616

**Published:** 2019-07-23

**Authors:** Efe Sezgin, Ping An, Cheryl A. Winkler

**Affiliations:** ^1^Laboratory of Nutrigenomics and Epidemiology, Izmir Institute of Technology, Urla, Turkey; ^2^Basic Research Laboratory, Center for Cancer Research, National Cancer Institute, Leidos Biomedical Research, Inc., Frederick National Laboratory for Cancer Research, Frederick, MD, United States

**Keywords:** cytomegalovirus, host genetics, viral pathogenesis, immune response, genetic epidemiology

## Abstract

Human cytomegalovirus (HCMV) is a ubiquitous herpes virus (human herpes virus 5) with the highest morbidity and mortality rates compared to other herpes viruses. Risk groups include very young, elderly, transplant recipient, and immunocompromised individuals. HCMV may cause retinitis, encephalitis, hepatitis, esophagitis, colitis, pneumonia, neonatal infection sequelae, inflammatory, and age-related diseases. With an arsenal of genes in its large genome dedicated to host immune evasion, HCMV can block intrinsic cellular defenses and interfere with cellular immune responses. HCMV also encodes chemokines, chemokine receptors, and cytokines. Therefore, genes involved in human viral defense mechanisms and those encoding proteins targeted by the CMV proteins are candidates for host control of CMV infection and reactivation. Although still few in number, host genetic studies are producing valuable insights into biological processes involved in HCMV pathogenesis and HCMV-related diseases. For example, genetic variants in the immunoglobulin GM light chain can influence the antibody responsiveness to CMV glycoprotein B and modify risk of HCMV-related diseases. Moreover, CMV infection following organ transplantation has been associated with variants in genes encoding toll-like receptors (TLRs), programmed death-1 (*PD-1*), and interleukin-12p40 (*IL-12B*). A KIR haplotype (2DS4+) is proposed to be protective for CMV activation among hematopoietic stem cell transplant patients. Polymorphisms in the interferon lambda 3/4 (*IFNL3/4*) region are shown to influence susceptibility to CMV replication among solid organ transplant patients. Interestingly, the *IFNL3/4* region is also associated with AIDS-related CMV retinitis susceptibility in HIV-infected patients. Likewise, interleukin-10 receptor 1 (*IL-10R1*) variants are shown to influence CMV retinitis development in patients with AIDS. Results from genome-wide association studies suggest a possible role for microtubule network and retinol metabolism in anti-CMV antibody response. Nevertheless, further genetic epidemiological studies with large cohorts, functional studies on the numerous HCMV genes, and immune response to chronic and latent states of infection that contribute to HCMV persistence are clearly necessary to elucidate the genetic mechanisms of CMV infection, reactivation, and pathogenesis.

## Introduction

Human cytomegalovirus (HCMV), also called human herpes virus 5 (HHV5), is a beta herpesvirus that belongs to the Herpesviridae family (Davison, 2007; Davison and Bhella, 2007; Liu and Zhou, 2007). HCMV exhibits broad cellular tropism, capable of infecting most cell types and organs. As an opportunistic pathogen, HCMV is ubiquitous with a global infection distribution and causes more morbidity and mortality compared to any other herpes virus (Wills et al., 2007). Major HCMV transmission routes include saliva, sexual contact, placental transfer, breast feeding, blood transfusion, solid organ transplantation, and hematopoietic stem cell transplantation (HSCT; Pass, 1985; Ho, 1990). The incidence of infection and prevalence increases progressively with age, reaching over 70% prevalence by age 70 in developed countries. The seroprevalence rates can be more than 90% among lower socioeconomic groups, men who have sex with men, and in developing countries (Pass, 1985; Ho, 1990; Stagno and Cloud, 1990; Razonable, 2005; Cannon, 2009; Beam and Razonable, 2012).

HCMV, with a double-stranded linear DNA genome ranging between 196 and 241 kbp (thousand base pairs), has the largest genome among the betaherpesviruses. The genome can encode over 160 gene products, a number much higher than other betaherpesviruses (Murphy et al., 2003a; Murphy et al., 2003b; Dolan et al., 2004). Only a subset of the 160 genes have roles in herpesvirus core function such as DNA replication, DNA encapsulation, and virion maturation, whereas the majority are involved in viral persistence, latency, diverse cellular tropism, and host immune response modulation, indicating complex interactions throughout HCMV co-evolution with its human host (Stern-Ginossar et al., 2012). For example, HCMV encodes homologs of cellular chemokines, chemokine receptors, and cytokines, which might contribute to immune evasion of infected host cells (McSharry et al., 2012).

The recognition of CMV as a medically important virus goes back to early 1930s when cytomegalic inclusion disease, a severe form of congenital CMV disease with an owl’s eye appearance of inclusion bodies in cells from multiple organs of the infants, was observed. By 1970s, the pathogenic organ disease and HCMV link was well established, and HCMV-like viruses were isolated from other mammals. Due to the high social and medical cost of congenital CMV disease (i.e., sensorineural hearing loss and other severe neurological injury), vaccine development is a high public health priority (Plotkin, 2004; Arvin et al., 2004). HCMV continued to draw increasing medical attention as an opportunistic infection in immunocompromised individuals receiving organ transplants and the elderly. Moreover, persistent HCMV infection has been demonstrated to accelerate immunosenescence also known as human immune aging (Koch et al., 2006; Koch et al., 2007; Pawelec et al., 2009; Wistuba-Hamprecht et al., 2013; Pawelec, 2014). The onset of the HIV epidemic and the concomitant increase in AIDS-related CMV infections led to the development of several antiviral drugs (Plotkin, 2004; Griffiths and Boeckh, 2007; Kotton, 2013; Shin et al., 2014; Vora et al., 2018). However, currently there is no protective vaccination, and viral resistance against available antiviral drugs necessitates continuing research and investment in better understanding of CMV pathogenesis (Plotkin, 2002; Schleiss et al., 2006; Heineman, 2007; Griffiths and Boeckh, 2007; Plotkin and Boppana, 2018).

**Figure 1:**
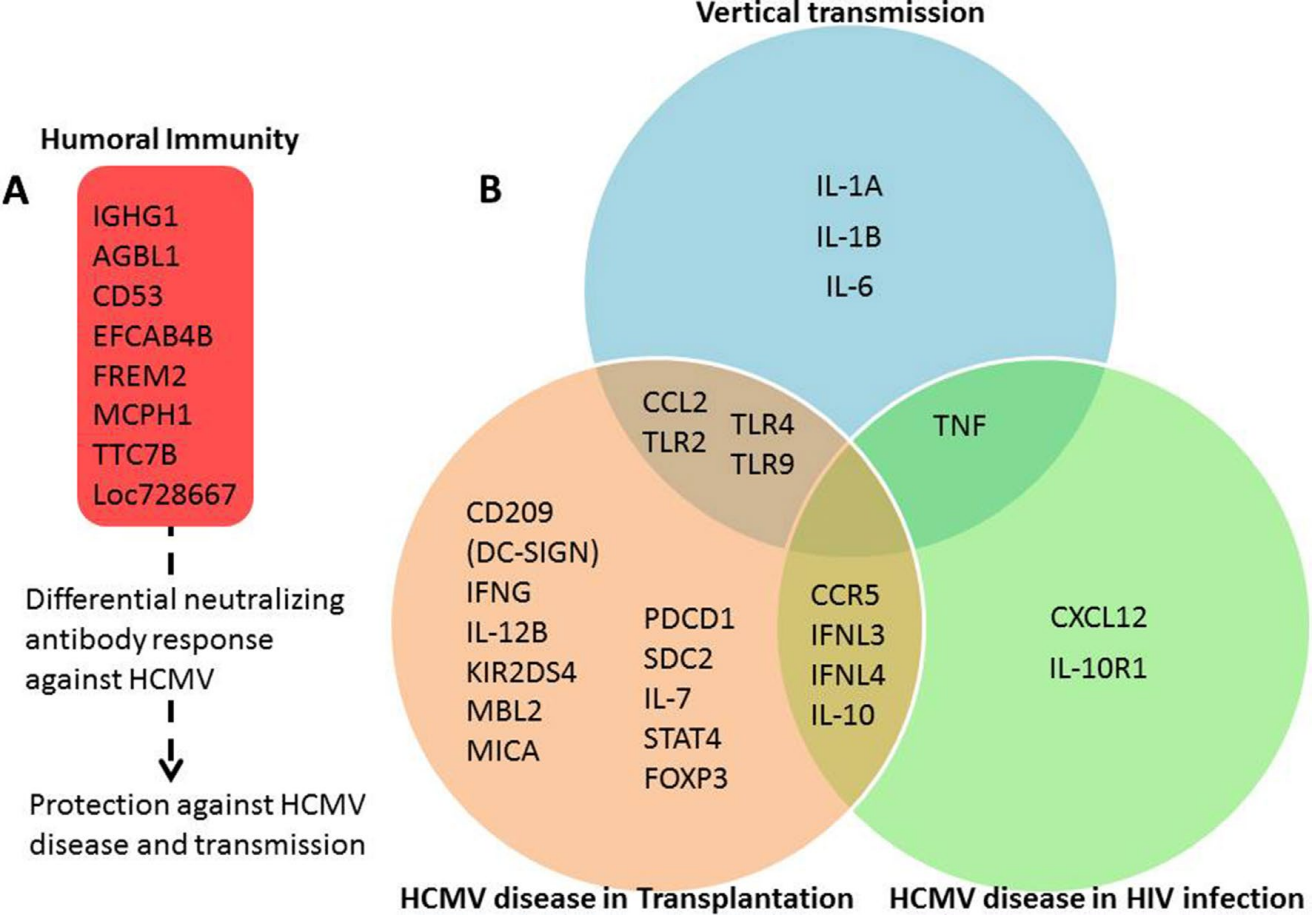
Human genes involved in response to HCMV and related diseases. Summary figure representing the interaction between host genes and HCMV in different phenotypic outcomes based on literature reports as listed and cited in **Table 1**. **(A)** Genes modulating humoral immunity response against HCMV. **(B)** Innate and adaptive immune response modulating genes unique to and shared between susceptibility to HCMV disease in transplantation, HIV infection, and vertical transmission categories. The grouping of genes among the phenotypic outcomes is deduced based on association results and related literature detailed in **Table 1**. “Vertical transmission” category is based on studies of transmission of HMCV from mother to fetus or newborn. “HCMV disease in transplantation” category includes results from solid organ and stem cell transplantation studies. “HCMV disease in HIV infection” category includes results from HIV-infected patients.

Most reviews in CMV literature focus on the viral and immune response aspects of the pathogenesis. However, host genetics of viral infection and pathogenesis can identify biological pathways that may lead to novel therapeutics. This review takes a different approach and aims to cover the current cumulative state of the knowledge in the host genetics of CMV pathogenesis in different risk groups. Different phenotypic outcomes of HCMV susceptibility are presented in the following sections. The details of genetic associations such as cohorts, odds ratios, P-values, and sample size are presented in **Table 1**. A summary figure of interactions between host genes and HCMV in different phenotypic outcomes based on literature reports is presented in **Figure 1**.

**Table 1:** Loci reported to be involved in HCMV and related disease susceptibility.

Phenotype	Gene	Variant/genotype	Location	Sample size	Model	Effect	P-value	Population	Study group	Ref.
**Humoral immunity**										
	IGHG1	GM 3	Exon	131	Recessive	Reduced anti-HCMV Glycoprotein B Immunoglobulin G (IgG) Antibody level	0.01	USA/Europeans	Upper Midwest Health Study	(Pandey et al., 2014c)
	IGHG1	GM 5	Exon	131	Recessive	Reduced anti-HCMV Glycoprotein B Immunoglobulin G (IgG) Antibody level	0.01	USA/Europeans	Upper Midwest Health Study	(Pandey et al., 2014c)
	AGBL1	rs2011905 -C Allele	Intron	2442	Allelic	Increased anti-CMV IgG titer	1.9 x 10^−6^	CVD-Finns	24–39 year old participants (GWAS)	(Kuparinen et al., 2012)
	CD53	rs2885805 -A allele	Intron	2442	Allelic	Decreased anti-CMV IgG titer	4.6 x 10^−6^	CVD-Finns	24–39 year old participants (GWAS)	(Kuparinen et al., 2012)
	EFCAB4B	rs4766152 -G allele	Intron	2442	Allelic	Decreased anti-CMV IgG titer	5.1 x 10^−6^	CVD-Finns	24–39 year old participants (GWAS)	(Kuparinen et al., 2012)
	FREM2	rs9594293 -A allele	Intron	2442	Allelic	Decreased anti-CMV IgG titer	6.8 x 10^−6^	CVD-Finns	24–39 year old participants (GWAS)	(Kuparinen et al., 2012)
	MCPH1	rs1707715 - G allele	Intron	2442	Allelic	Increased anti-CMV IgG titer	7.8 x 10^−6^	CVD-Finns	24–39 year old participants (GWAS)	(Kuparinen et al., 2012)
	TTC7B	rs1779382 - A allele	Intron	2442	Allelic	Increased anti-CMV IgG titer	9.1 x 10^−6^	CVD-Finns	24–39 year old participants (GWAS)	(Kuparinen et al., 2012)
	LOC728667 or LINC00596	rs1288981 - T allele	Between genes	1300	Allelic	Decreased IgG antibody response against HCMV	8.2 x 10^−7^	Mexican Americans in the San Antonio Family Study	16–94 year old participants (GWAS)	(Rubicz et al., 2015)
**Cancer susceptibility**										
	IGHG1	GM 3	–	253 (120 case, 133 control)	Recessive	Increased risk of Glioma	0.04	Portugal/European	Patients with glioma	(Pandey et al., 2014b)
	IGHG1	GM 3/ GM 17	–	253 (120 case, 133 control)	Genotype	Increased risk of Glioma	0.02	Portugal/European	Patients with glioma	(Pandey et al., 2014b)
	IGHG1	GM 3	–	258 case–control pair	Additive	Increased breast cancer risk	0.01	Brazil/Europeans	Patients with invasive breast cancer	(Pandey et al., 2012)
	IGHG1	GM 3	–	251	Additive and Recessive	Reduced anti-HCMV Glycoprotein B Immunoglobulin G (IgG) Antibody level	0.01–0.03	Brazil/Europeans	Breast cancer free controls	(Pandey et al., 2016)
**HCMV disease in transplantation**										
***Solid organ transplantation***										
	TLR2	R753Q (rs5743708, 2258 G > A)	Exon	737 (92 cases, 645 controls)	Recessive	Increased risk of CMV disease after liver transplantation	0.003	USA/Europeans	Patients who received liver transplantation at the Mayo Clinic in Rochester, Minnesota	(Kang et al., 2012)
	TLR2	R753Q (rs5743708, 2258 G > A)	Exon	92	Recessive	Increased HCMV viral load and risk of CMV disease after liver transplantation	0.003–0.04	USA/Europeans	Patients who received liver transplantation at the Mayo Clinic in Rochester, Minnesota	(Kijpittayarit et al., 2007)
	TLR4	D299G (rs4986790) or T399I (rs498679)	Exon	245	Presence of any of the variants	Increased risk of primary HCMV infection and disease	0.02	Spain	Patients who received kidney or kidney-pancreas transplantation	(Cervera et al., 2007)
	TLR9	rs5743836 – TT genotype	Upstream	315	Recessive	Lower incidence of HCMV infection	0.035	Spain	Patients received kidney transplantation in the OPERA study	(Fernandez-Ruiz et al., 2015)
	DC-SIGN	rs735240 – GG genotype	Upstream	315	Recessive	Higher incidence of HCMV infection	0.008	Spain	Patients received kidney transplantation in the OPERA study	(Fernandez-Ruiz et al., 2015)
	MBL2	Biochemical deficiency/Null	–	16	Recessive	Increased risk of HCMV infection	0.005	Univ. Hospital, Lausanne, CH	Patients who received kidney transplantation	(Manuel et al., 2007)
	PDCD1/ PD-1	rs11568821 - G allele	Intron	469	Recessive	Increased risk of HCMV infection	0.01	Univ. Hospital, Tours, France	Patients who received kidney transplantation	(Hoffmann et al., 2010)
	PDCD1/ PD-1	rs11568821 -A allele	Intron	1119	Dominant	Improved kidney graft survival recipients receiving grafts from CMV-positive donors	0.002	Univ. Hospital, Tours, France	Patients who received kidney transplantation	(Forconi et al., 2017)
	PDCD1/ PD-1	rs11568821 -A allele	Intron	181	Dominant	Improved lung graft survival recipients receiving grafts from CMV-positive donors	0.006	University Hospital of Tours, France	Patients who received lung transplantation	(Forconi et al., 2017)
	IFNG	+874T/A (rs2430561) -TT genotype	Intron	170	Genotypic	High level of viremia and HCMV disease	0.001	USA/Europeans	Patients who received lung transplantation	(Mitsani et al., 2011)
	IFNG	+874T/A (rs2430561) -AA genotype		247	Genotypic	Increased risk of HCMV infection and disease	0.01	USA/Hispanics	Patients who received kidney transplantation	(Vu et al., 2014)
	IFNL3/ IFNL4	rs8099917 -G allele	5’ Upstream region	38 (17 with CMV replication, 21 no CMV replication)	Dominant	Reduced HCMV replication	0.04	University of Alberta, Canada	Patients who received solid organ transplantation	(Egli et al., 2014)
	IFNL3/ IFNL4	rs368234815 -G allele	CpG region, 5’ Upstream	840	Recessive	Increased risk of HCMV replication and disease	0.05	Europeans/Swiss Transplant Cohort Study	Patients who received solid organ transplantation	(Manuel et al., 2015)
	IFNL3/ IFNL4/IL28B	rs12979860 – T allele	Intro	315	Allelic	Lower incidence of HCMV infection	0.03	Spain	Patients received kidney transplantation in the OPERA study	(Fernandez-Ruiz et al., 2015)
	IL-10	-1082A/G -AA genotype	5’ Upstream	408	Genotypic	Reduced incidence of HCMV infection	0.03	Finland	Patients who received kidney transplantation	(Alakulppi et al., 2006)
	IL-12B	rs3212227 -C allele (IL-12p40)	3′-untransla-ted region	469	Allelic	Increased risk of HCMV infection	0.04	University Hospital of Tours, France	Patients who received kidney transplantation	(Hoffmann et al., 2008)
	MICA	rs2596538	5’ Upstream region	181	Allelic	Protective against HCMV infection and disease	0.001	University Hospital Essen, Germany	Patients who received kidney transplantation	(Rohn et al., 2018)
***Hematopoietic transplantation ***										
	IFNL3/ IFNL4	rs12979860 -T allele	Intron	151	Genotypic	Protective against HCMV infection	0.04	University Clinic Hospital of Valencia, Spain	Patients who received allogeneic stem cell transplantation	(Bravo et al., 2014)
	IFNL3/ IFNL4	rs12979860 -T allele	Intron	142	Recessive	Protective against HCMV infection	0.05	University Clinic Hospital of Valencia, Spain	Patients who received allogeneic stem cell transplantation	(Corrales et al., 2017)
	IFNL3/ IFNL4	rs12979860 -T allele; rs368234815-ΔG allele	Intron; Exon	99	Compound Genotypic	Increased risk of HCMV activation	< 0.05	Italian cohorts	Patients who received allogeneic stem cell transplantation	(Annibali et al., 2018)
	IL-10	rs1800893 -G allele	5’ Upstream	154 (83 HCMV activation, 71 control)	Additive	Increased risk of HCMV disease	0.009	Multinational/Europeans	Patients who received allogeneic stem cell transplantation	(Loeffler et al., 2006)
	IL-10	rs1800896 -G allele	5’ Upstream	154 (83 HCMV activation, 71 control)	Additive	Increased risk of HCMV disease	0.001	Multinational/Europeans	Patients who received allogeneic stem cell transplantation	(Loeffler et al., 2006)
	IL-10	rs1878672 -G allele	Intron	154 (83 HCMV activation, 71 control)	Additive	Increased risk of HCMV disease	0.003	Multinational/Europeans	Patients who received allogeneic stem cell transplantation	(Loeffler et al., 2006)
	IL-10	rs3024492 -T allele	Intron	154 (83 HCMV activation, 71 control)	Additive	Increased risk of HCMV disease	0.04	Multinational/Europeans	Patients who received allogeneic stem cell transplantation	(Loeffler et al., 2006)
	IL-7	rs6897932 -T allele	Exon	460	Genotypic	Increased risk of HCMV infection	0.007	Copenhagen University, Denmark	Patients who received allogeneic stem cell transplantation	(Kielsen et al., 2018)
	CCL2 (MCP1)	rs1024611 -T allele	5’ Upstream	154 (83 HCMV activation, 71 control)	Additive	Increased risk of HCMV reactivation	0.03	Multinational/Europeans	Patients who received allogeneic stem cell transplantation	(Loeffler et al., 2006)
	CCL2 (MCP1)	rs13900 -T allele	Exon	154 (83 HCMV activation, 71 control)	Additive	Increased risk of HCMV reactivation	0.02	Multinational/Europeans	Patients who received allogeneic stem cell transplantation	(Loeffler et al., 2006)
	CCR5	rs17141079 -T allele	Intron	154 (83 HCMV activation, 71 control)	Additive	Increased risk of HCMV disease	0.02	Multinational/Europeans	Patients who received allogeneic stem cell transplantation	(Loeffler et al., 2006)
	CCR5	rs1800023 -G allele	5’ Upstream	154 (83 HCMV activation, 71 control)	Additive	Increased risk of HCMV disease	0.01	Multinational/Europeans	Patients who received allogeneic stem cell transplantation	(Loeffler et al., 2006)
	CCR5	rs1800023 -A allele	5’ Upstream	102	Additive, Recessive	Increased CMV DNAemia and DNA peak	0.02, 0.05	Spanish cohort	Patients who received allogeneic stem cell transplantation	(Corrales et al., 2015)
	CCR5	rs2734648 -T allele	5’ Upstream	154 (83 HCMV activation, 71 control)	Additive	Increased risk of HCMV disease	0.01	Multinational/Europeans	Patients who received allogeneic stem cell transplantation	(Loeffler et al., 2006)
	CD209 (DC-SIGN)	rs2287886 -T allele	5’ Upstream	194 (70 HCMV reactivation, 59 HCMV disease, 65 control)	Allelic	Increased risk of development of HCMV reactivation and disease	0.003	Germany/Europeans	Patients who received allogeneic stem cell transplantation	(Mezger et al., 2008)
	CD209 (DC-SIGN)	rs735240 -A allele	5’ Upstream	194 (70 HCMV reactivation, 59 HCMV disease, 65 control)	Allelic	Increased risk of development of HCMV reactivation and disease	0.01	Germany/Europeans	Patients who received allogeneic stem cell transplantation	(Mezger et al., 2008)
	SDC2	rs1042381 -T allele	Exon	194 (70 HCMV reactivation, 59 HCMV disease, 65 control)	Allelic	Increased risk of development of HCMV reactivation and disease	0.04	Germany/Europeans	Patients who received allogeneic stem cell transplantation	(Mezger et al., 2008)
	KIR2DS4	KIR1D+ (deletion) haplotype	–	165	Recessive	Increased risk of HCMV reactivation	0.002	Chinese cohort	Patients with hematopoietic stem cell transplantation	(Wu et al., 2016)
	STAT4	Rs7574865 -T allele	Intron	161	Recessive	Increased risk of HCMV infection	0.01	Seoul National University, Korea	Patients with hematopoietic stem cell transplantation	(Wun et al., 2017)
	FOXP3	Rs3761548 -C allele	Intron	171	Recessive	Increased risk of HCMV infection	0.01	Seoul National University, Korea	Patients with hematopoietic stem cell transplantation	(Piao et al., 2016)
**HCMV disease in HIV infection**										
	IL-10	rs3024496 -C Allele	3’ UTR	534 (110 cases, 424 controls)	Dominant	Susceptible to CMV-Retinitis	0.05	USA/African Americans	Patients with AIDS	(Sezgin et al., 2010)
	IL-10	rs3024500 -C Allele	3’ UTR	534 (110 cases, 424 controls)	Dominant	Susceptible to CMV-Retinitis	0.02	USA/African Americans	Patients with AIDS	(Sezgin et al., 2010)
	IL-10R1 (IL10RA)	rs2228055 -G allele	Exon	750 (200 cases, 550 controls)	Haplotypic	Susceptible to CMV-Retinitis	0.04	USA/Europeans	Patients with AIDS	(Sezgin et al., 2010)
	IL-10R1 (IL10RA)	rs2229114 -T allele	Exon	750 (200 cases, 550 controls)	Allelic	Protective against CMV-Retinitis	0.03	USA/Europeans	Patients with AIDS	(Sezgin et al., 2010)
	CCR5	rs1799988	5’ UTR, CCR5 P1 Promoter Haplotype	117	Haplotypic	Increased risk of CMV-Retinitis progression	0.007	USA/African Americans	Patients with AIDS	(Sezgin et al., 2011)
	CCR5	rs1799988	5’ UTR, CCR5 P1 Promoter Haplotype	203	Haplotypic	Increased risk of mortality in patients with CMV-Retinitis	0.05	USA/African Americans	Patients with AIDS	(Sezgin et al., 2011)
	CXCL12 (SDF1)	rs1801157 -A allele	3′-untranslat-ed region	117	Dominant	Increased risk of CMV-Retinitis progression	0.008	USA/African Americans	Patients with AIDS	(Sezgin et al., 2011)
	TNF	4-1-G-2-2-1	5’ Upstream	222 (52 CMV-retinitis, 170 control)	Haplotypic	Susceptible to CMV-Retinitis	0.03	Brazilian cohort (mixed race)	Patients with AIDS	(Deghaide et al., 2009)
	TNF	TNFc1	5’ Upstream	222 (52 CMV-retinitis, 170 control)	Allelic	Protective against CMV-Retinitis	0.04	Brazilian cohort (mixed race)	Patients with AIDS	(Deghaide et al., 2009)
	IFNL3/ IFNL4	rs368234815 -G allele	CpG region, 5’ Upstream	1134	Recessive	Increased risk of CMV-retinitis	0.007	Europeans/Swiss HIV Cohort Study	Patients with HIV infection	(Bibert et al., 2014)
**HCMV disease in vertical transmission**										
	TLR2	rs1898830 -AG genotype	Intron	170 (87 case, 83 control)	Genotypic	decreased risk of congenital HCMV infection	0.03	Japanese cohort	Children with congenital HCMV infection	(Taniguchi et al., 2013)
	TLR2	rs1898830 -GG genotype	Intron	83 (33 case, 50 control)	Recessive	Protection against HCMV transmission	0.008	Israeli cohort	Pregnant women with HCMV infection	(Eldar-Yedidia et al., 2017)
	TLR2	rs3804100 (1350 T > C) -CC genotype	Exon	170 (87 case, 83 control)	Genotypic	Increased risk of congenital HCMV infection	0.01	Japanese cohort	Children with congenital HCMV infection	(Taniguchi et al., 2013)
	TLR2	Arg677Trp (rs121917864, 2029 C > T)	Exon	229 (151 case, 78 control)	Allelic	Reduced risk of infection only in adults	< 0.001	Polish cohort	HCMV-infected children and adults	(Jablonska et al., 2014)
	TLR4	D299G (rs4986790)	Exon	229 (151 case, 78 control)	Allelic	Reduced risk of infection only in adults	0.02	Polish cohort	HCMV-infected children and adults	(Jablonska et al., 2014)
	TLR2	R753Q (rs5743708, 2258 G > A)	Exon	51 (20 case, 30 control)	Genotypic	increased risk of congenital HCMV infection	0.02	Polish cohort	HCMV-infected fetuses and neonates	(Wujcicka et al., 2017a)
	TLR9	rs352140 (2848 G > A) - A allele	Exon (synonymous change)	131 (66 case, 65 control)	Dominant	Reduced risk of HCMV infection in pregnant women	0.03	Polish cohort	HCMV-infected pregnant women	(Wujcicka et al., 2017b)
	TLR9	rs352140 (2848 G > A) -TC genotype	Exon (synonymous change)	142 (72 case, 70 control)	Genotypic	Increased risk of HCMV infection in infants	0.02	Polish cohort	Congenitally HCMV-infected infants	(Paradowska et al., 2016)
	TLR9	Rs187084 (-1486 T > C) -C allele	Upstream	142 (72 case, 70 control)	Dominant	Increased risk of HCMV infection in infants	0.02	Polish cohort	Congenitally HCMV-infected infants	(Paradowska et al., 2016)
	IL1A	-889 C > T	5’ Upstream	51 (20 case, 31 control)	Allelic	increased risk of congenital HCMV infection and onset of related symptoms	< 0.0001	Polish Mother’s Memorial Hospital, Poland	Fetuses and Neonates with HCMV infection	(Wujcicka et al., 2017c)
	IL1A	-889 C > T	5’ Upstream	129 (65 case, 64 control)	Recessive	Decreased risk of HCMV infection	0.05	Polish Mother’s Memorial Hospital, Poland	Pregnant women	(Wujcicka et al., 2017d)
	IL1B	rs1143634 (+3954 C > T) -T allele	Exon	51 (20 case, 31 control)	Allelic	increased risk of congenital HCMV infection and onset of related symptoms	< 0.0001	Polish Mother’s Memorial Hospital, Poland	Fetuses and Neonates with HCMV infection	(Wujcicka et al., 2017c)
	IL1B	rs16944 -T allele	5’ Upstream	470 (72 case, 398 control)	Genotypic	increased risk of intrauterine HCMV infection	0.03	Infants enrolled at the Children’s Memorial Health Institute in Warsaw, Poland	Infants with HCMV infection	(Kasztelewicz et al., 2017)
	IL6	-174 G > C	5’ Upstream	129 (65 case, 64 control)	Recessive	Decreased risk of HCMV infection in their offspring	0.02	Polish Mother’s Memorial Hospital, Poland	Pregnant women	(Wujcicka et al., 2017d)
	CCL2 (MCP1)	rs13900 -T allele	Exon	470 (72 case, 398 control)	Genotypic	increased risk of hearing loss at birth	0.03	Infants enrolled at the Children’s Memorial Health Institute in Warsaw, Poland	Infants with HCMV infection	(Kasztelewicz et al., 2017)
	TNF	rs1799964 -T allele	5’ Upstream	470 (72 case, 398 control)	Genotypic	increased risk of intrauterine HCMV infection	0.03	Infants enrolled at the Children’s Memorial Health Institute in Warsaw, Poland	Infants with HCMV infection	(Kasztelewicz et al., 2017)

All values and information are based on the cited paper. Whenever case–control status or disease-specific divisions of patients were available, they are reported under the sample size column.

IGHG1: Immunoglobulin GM γ chain marker.

CVD-Finns: Cardiovascular risks in young Finns study.

## Host Genetics of Humoral Immunity to HCMV

HCMV immune human sera contain neutralizing antibodies against principal CMV envelope proteins (such as gB), tegument phosphoprotein pp150 (UL32), and nonstructural DNA binding phosphoprotein pp52 (UL44) (Landini, 1993). Humoral immunity to CMV can be protective against blood-borne spread of virus, transplacental transmission, and CMV acquisition and disease (Jonjic et al., 1994; Plotkin et al., 1994; Schoppel et al., 1998; Fields et al., 2001; Schleiss et al., 2004). There is differential response to CMV exposure, and not everyone exposed to HCMV develops a CMV-related disease, suggesting a possible role for host genetic variation in antibody response to HCMV.

Immunoglobulins, also known as antibodies, constitute a critical part of the humoral immune response by specifically recognizing and binding to particular antigens. Immunoglobulin G is the most common type of antibody in circulation. Variation in genes that code for immunoglobulin (Ig) GM (gamma marker) creates several alleles (also referred as allotypes), (i.e., GM 3 and GM 17) with different binding affinities to antigens such as HCMV glycoprotein B (gB). The effect of genetic variation in humoral immunity on susceptibility to HCMV disease has been suggested by the studies that focus on association between immunoglobulin (Ig) GM allotype variation and HCMV antibody response. Pandey et al. reported a significant effect of immunoglobulin GM genotypes on antibody responsiveness to HCMV glycoprotein B (gB) (Pandey, 2014a). The study showed significant differences in antibody response to HCMV between GM 3 and GM 17 alleles. HCMV codes for a Fc gamma receptor (FcgR)-like protein (coded by the HCMV *RL13* gene) that can bind to the anti-HCMV IgG antibody, thus reducing the number of free anti-HCMV antibodies in circulation, and giving survival advantage to the virus. The GM 3 allele has higher affinity to HCMV FcgR-like protein (through bipolar bridging) compared to the GM 17 allele, leaving lower concentration of free anti-HCMV gB antibodies circulating in the system. They also drew attention to B-cell-mediated antigen processing/presentation pathway as an alternative mechanism underlying GM allotypes’ differential responsiveness to HCMV gB. One of the strategies that HCMV has evolved for evading host immunosurveillance involves generating proteins with similar functional properties to the Fcγ receptor for IgG (FcγR). FcγR interferes with the anti-HCMV IgG antibody’s binding to the virus, thus giving survival advantage to HCMV against antibody-dependent cellular cytotoxicity, antibody-dependent cellular phagocytosis, and antibody-dependent complement-dependent cytotoxicity (Atalay et al., 2002; Namboodiri and Pandey, 2011).

A major issue in genetic epidemiology studies of antibody variation is that most of the genetic variation is ethnic and population specific. For example, GM 3 is rare among people of African descent. Moreover, the genetic background influencing overall immune response will be different between races and populations. Therefore replication and translation of results from one study to another is not always possible. Ethnic and population-specific genetic variation can also lead to hidden population stratification even in the same country, which is major factor confounding the genetic association results. Given the complex genetic nature of humoral immune response to HCMV, much larger studies with more balanced case and control groups are needed to test the GM associations with HCMV response.

## Host Genetics of HCMV in Cancers

HCMV is not considered an oncogenic virus; however, HCMV viral DNA, RNA, and protein have been frequently found in neoplastic tissues including gliomas, breast cancer, and neuroblastoma (Cobbs et al., 2002; Harkins et al., 2010; Cobbs, 2011; Taher et al., 2013; Wolmer-Solberg et al., 2013). Moreover, HCMV infection leads to changes in cell physiology, tumor microenvironment, inhibition of apoptosis, and evasion from immune detection that are characteristics of cancer development (Hanahan and Weinberg, 2011). Not all HCMV-infected individuals develop cancers, suggesting that host genetics of HCMV response may influence HCMV-mediated cancer risk. Indeed, studies with glioma patient cohorts showed a modulating effect of GM alleles on the risk of gliomas, where the IgGM 3 homozygotes were over twice, and the GM 3/17 heterozygotes were over three times as likely to develop glioma (Pandey, 2014a; Pandey et al., 2014b). The magnitude of antibody responsiveness to HCMV glycoprotein B (gB) has also been implicated in breast cancer susceptibility. The GM 3 allele of IgG1 was reported to be significantly associated with increased susceptibility to breast cancer in Caucasian subjects from Brazil; however, the association was not significant in other population groups (Pandey et al., 2012). In a follow-up study, breast cancer-free individuals had significantly higher levels of anti-gB IgG antibodies than patients with breast cancer; however, there was interindividual and interethnic variability in the magnitude of antibody response, and interactions with other genes of the immune system were apparent (Pandey et al., 2016). Functional studies indicate that the binding of HCMV FcgR-like protein to GM 17 allele expressing IgG antibodies was significantly higher than GM 3 expressing antibodies, providing possible mechanistic insights for increased breast cancer risk in some HCMV-infected patients (Pandey et al., 2017).

Significant association of rare GM genotypes with neuroblastoma, a rare extracranial solid tumor, has been reported (Morell et al., 1977), but the mechanism underlying this association is still not clear. These uncommon GM genotypes include the GM 3, the allele with high affinity to HCMV TRL11/IRL11-encoded FcγR. Reports documenting early and late HCMV protein expression in primary neuroblastomas and neuroblastoma xenografts suggest either infection and transformation of neuroblastoma progenitor cells or direct infection of neuroblastoma cells and disturbance of intracellular pathways leading to neoplasms (Wolmer-Solberg et al., 2013). The mechanisms underlying the increased HCMV associated cancer risk with the GM 3 allele may also be involved in neuroblastoma cases as well.

Independent cohort and functional studies make a case for significant influence of host genetic variation in humoral immunity on response to HCMV disease (**Table 1**, **Figure 1**). However, increased cancer risk associated with increased HCMV susceptibility is still a hypothesis to be tested. Clearly, larger multi-ethnic, multi-cohort host genetic, and comprehensive functional studies are needed to uncover the host genetics of humoral immunity to HCMV and HCMV associated cancers.

## Host Genetics of HCMV Disease in Transplant Patients

HCMV is a common opportunistic infection among immunocompromised individuals. Individuals are maximally immunocompromised due to use of immunosuppressants during solid organ or HSCT procedures and thus are prone to HCMV reactivation (of the latent virus), primary infection, and reinfection. HCMV infections can cause severe morbidity and transplant failure, which frequently results in extended hospital stay and substantially higher cost of care (Falagas et al., 1997; Kim et al., 2000; Ljungman et al., 2002; Ramanan and Razonable, 2013). Transplantations from a seropositive individual to a seronegative individual (R-/D+) pose the greatest risk for HCMV-associated disease in the transplant recipient patients (Cope et al., 1997; Lowance et al., 1999). Therefore, determining the serologic status of the recipient and donor is important in assessing the risk of HCMV-associated disease. However, it can be hard to find serostatus matched donor and recipients, and even serostatus matching does not completely eliminate HCMV-associated morbidity.

Coordinated innate and adaptive immune response is crucial for control of HCMV infection in immunocompromised transplant recipients. Whereas innate interferon (IFN) and natural killer (NK) cell responses are important in immediate control of CMV infection, adaptive T cell immune responses are important in both active infection and reactivation control phases (Crough and Khanna, 2009; Zelini et al., 2010; Reddehase, 2013; Muntasell et al., 2013). To reduce the number of HCMV-associated adverse outcomes and better identify transplant patients for HCMV prophylaxis, several candidate innate and adaptive immune-related gene studies have been conducted (**Table 1**).

### Solid Transplantation Studies

A family of transmembrane proteins, the Toll-like receptors (TLRs), are part of the innate immune system and play crucial roles in the activation of the immune system by regulating the production of antiviral peptides and inflammatory cytokines against viral replication. The detection of CMV envelope glycoproteins B (gB) and H (gH) by TLR2 leads to nuclear factor-kB (NF-kB) activation and cytokine secretion against CMV (Boehme et al., 2006). Clinical studies showed that polymorphisms in TLR-2 (Kijpittayarit et al., 2007; Kang et al., 2012), TLR-4, TLR-9 (Fernandez-Ruiz et al., 2015), and mannose binding lectin (Cervera et al., 2007; Manuel et al., 2007) can be associated with increased risk of HCMV infection and disease after transplantation.

Genetic variants of MICA (major histocompatibility complex class I chain-related protein A) and its activating receptor NKG2D (natural killer group 2 member D) receptor may be associated with HCMV disease risk among kidney transplant patients. A candidate gene association study identified a regulatory MICA variant (rs2596538) in the kidney donors that can be a protective prognostic determinant for CMV disease. This functional variant was able to predict the development of CMV infection and disease during the first year after kidney transplantation (**Table 1**: Rohn et al., 2018). Membrane-associated molecule PD-1 (programmed death-1) regulates immune responses by inhibiting T cell receptor signaling, cytokine production in effector T cells, and expression on regulatory T cells (Sharpe et al., 2007; Franceschini et al., 2009). PD-1’s expression also correlates with CMV viremia in transplant patients (Sester et al., 2008). An upstream regulatory region variant (rs11568821) that impairs the function of PD-1 (also called PD-1.3) has been investigated in HCMV infection in kidney and lung graft recipients. The PD-1.3 variant has been shown to be associated with higher risk of HCMV infection (Hoffmann et al., 2010) and lung allograft survival in recipients from HCMV-positive donors (Forconi et al., 2017). Dendritic cell-specific ICAM 3-grabbing nonintegrin (DC-SIGN) variants were also reported to be associated with higher incidence of HCMV infection in kidney transplant patients (Fernandez-Ruiz et al., 2015).

Cytokines, signaling molecules of the immune system, regulate pro-inflammatory and anti-inflammatory responses, and play important roles in antiviral response. Cytokines also play a role in HCMV infection, reactivation, and disease (Asanuma et al., 1995; Zeevi et al., 1999; Faist et al., 2010; van de Berg et al., 2010; Biron and Tarrio, 2015; Nabekura and Lanier, 2016). Studies of functional gene polymorphisms in pro-inflammatory and anti-inflammatory cytokines with HCMV disease identified IFNG (interferon-gamma) +874 A/T polymorphism as a risk factor for HCMV disease in kidney and lung transplant patients, where the +874 A allele, a low IFNG producer (reduced gene expression), is associated with increased risk for HCMV infection and disease after organ transplantation (Mitsani et al., 2011; Vu et al., 2014). In a Finnish renal transplant cohort, the donor interleukin-10 (IL-10) gene polymorphism −1082AA was observed to influence HCMV infection risk. Recipient IL-10, IL-6, and IFNG polymorphisms also show significant associations with HCMV reactivation and disease risk (Alakulppi et al., 2006). A possible association between IL-12p40 gene polymorphisms in the recipient and high risk of HCMV infection was also reported after kidney transplantation (Hoffmann et al., 2008).

Type III interferon, also called interferon lambda (INFL3 or formerly IL-28B), has gained much attention as an important viral response element in recent years (Kotenko, 2011; Hayes et al., 2012). In a cohort of solid organ transplant patients from Alberta, a functional single nucleotide polymorphism (SNP) (rs8099917) associated with lower INFL3 (IL-28B) expression during CMV infection, but higher IFN-stimulated gene expression showed a protective effect against CMV replication (Egli et al., 2014). A follow-up Swiss Transplant Cohort study compared the cumulative incidence of CMV replication between patients with different TT/-G (rs368234815) genotype in the CpG region upstream of IFNL3. Patients with the –G/–G genotype had higher cumulative incidence of CMV replication. The study suggest that IFNL3 TT/-G (rs368234815) variant can be a CMV replication controller, particularly in patients not receiving antiviral prophylaxis (Manuel et al., 2015). Fernandez-Ruiz et al. (2015) reported a lower incidence of HCMV infections among kidney transplant patients with IL28B (IFNL3) rs12979860-T allele.

### Hematopoietic Transplantation Studies

Although still few in number, host genetics of HCMV susceptibility among HSCT cases have also been investigated (**Table 1**). Similar to solid organ transplant studies, several candidate innate and adaptive immunity genes have been examined. Results from solid organ transplantation studies stimulated cytokine and interferon research in HCMV disease in stem cell transplant settings. In a comprehensive immunogenetic study, allogeneic stem cell transplant patients with HCMV reactivation (DNAemia), patients with HCMV disease, and patients without HCMV reactivation were examined (Loeffler et al., 2006). Polymorphisms in the CCR5, IL-10, and MCP1 were observed to contribute to HCMV reactivation and disease after allogeneic stem cell transplantation (Loeffler et al., 2006; Corrales et al., 2015). In a follow-up study, this research group extended their investigation and observed a significant association between promoter region variants, which influenced the expression levels of DC-SIGN on dendritic cells, and increased risk of development of HCMV reactivation and disease (Mezger et al., 2008). Protective effects of the INFL3 rs12979860 C/T polymorphism against CMV infection (Bravo et al., 2014; Corrales et al., 2017) and INFL3 rs12979860 IFNL4 rs368234815 compound genotype against HCMV reactivation (Annibali et al., 2018) in the allogeneic stem cell transplant setting were also reported. Killer immunoglobulin-like receptors (KIR) are cell surface receptors found on NK and certain T cells. The activating and inhibitory signals are transmitted to NK cells through KIR proteins, by which NK cells respond quickly to infections. In a Chinese cohort HLA-matched HSCT patients, patients receiving HSCT from donors with heterozygote 2DS4+/1D+ KIR haplotype showed at least 20% less CMV reactivation compared to donors with other haplotypes (Wu et al., 2016). This observation suggests that donor KIR haplotype should be evaluated for HLA-matched HSCT cases. More recently functional genetic variants in *FOXP3* (Piao et al., 2016), *STAT4* (Wun et al., 2017), and *IL-7*(Kielsen et al., 2018) are reported to influence HCMV infection after HSCT in independent cohort studies.

Host genetics of HCMV disease in transplant patients provide hints towards promising genetic markers to predict CMV viremia (**Table 1**, **Figure 1**). However, these studies are complicated due to several factors, including donor and recipient serostatus, type and dose of immunosuppressive drugs used, and ethnicity of the patient cohort. These factors also lead to significant heterogeneity among the studies confounding the efficacy of a meta-analysis of the individual studies to reach a consensus on securely identified markers and causal variants. The transplant community is still far from developing well accepted genetic markers for personalized CMV approaches (such as decisions on prophylactic and preemptive therapies) to be used in the clinic.

## Host Genetics of HCMV Disease in HIV-Infected Patients

Patients with HIV infection are another group of immunocompromised individuals that are at high risk for CMV disease. Before the use of ART (antiretroviral therapy), up to 40% of adults with AIDS developed CMV disease (Gallant et al., 1992). Although the incidence of CMV infection has declined dramatically, new cases continue to occur (Sezgin et al., 2018). Among HIV-infected patients the risk of CMV disease is linked to CD4+ T-cell counts. The most common CMV disease among patients with uncontrolled HIV infection is CMV-Retinitis that may develop when CD4+ T-cell counts drop below 50–100 CD4+ cells/µl (Jabs, 1995; Dunn and Jabs, 1995; Heiden et al., 2007). While some patients with low CD4+ T-cell counts remain asymptomatic, others can progress to CMV disease rather quickly. Host genetic variants in genes involved in regulation of innate and adaptive immune responses may have a role in this differential susceptibility to CMV among HIV-infected patients as they have in other immunocompromised groups such as transplant patients.

A comprehensive candidate gene study on host genetics of CMV-Retinitis among HIV-infected patients was conducted in Longitudinal Studies of Ocular Complications of AIDS (LSOCA) cohort (**Table 1**). The study showed that human interleukin-10 receptor (IL-10R1) variants that potentially interfere with IL-10 binding and signal transduction can influence CMV-Retinitis occurrence in European Americans (Sezgin et al., 2010). The same study also suggested a possible role of IL-10 variants on CMV-Retinitis risk among African Americans (Sezgin et al., 2010). In a follow-up study of the same cohort, cytokine and cytokine receptor [*CCR5* and stromal derived factor (*SDF-1*)] genetic variants have been observed to influence retinitis progression (Sezgin et al., 2011). In a different cohort study, *TNF* polymorphisms were also linked to susceptibility to CMV retinitis in white patients, though with rather small sample size (Deghaide et al., 2009). In a large Swiss HIV Cohort Study, the effect of *IFNL3* TT/-G substitution, the variant that increased susceptibility to CMV replication in transplant patients (Manuel et al., 2015), was also shown to be associated with higher risk of CMV retinitis (Bibert et al., 2014).

Although subject to complex confounding factors and high false discovery rates, host candidate gene studies of immunocompromised groups cumulatively indicate possible effects of innate and adaptive immune gene variants on CMV disease (**Table 1**, **Figure 1**). More studies should be designed to replicate and validate these results.

## Host Genetics of Vertical HCMV Transmission

Vertical transmission of HCMV from mother to fetus or newborn is common and plays an important role in maintaining infection in the population (Stagno et al., 1982a; Whitley, 2004). Prenatal infection rates are highest in low income countries or low socioeconomic populations, where risk of maternal seropositivity is also high (Stagno et al., 1982b; Stagno et al., 1982c). Recurrent and primary HCMV infection during pregnancy can cause congenital infection of the newborn and may lead to severe clinical complications such as hearing defects, birth defects, and irreversible neurodevelopmental sequelae (Boppana et al., 1992; Boppana et al., 1999; Gaytant et al., 2002).

Host candidate genetic studies of congenital HCMV infection mainly have focused on innate immune system, such as TLRs and Mannan-binding lectins, and cytokine genes (**Table 1**, **Figure 1**). In children with congenital HCMV disease, the TLR2 1350 T > C variant (rs3804100) was reported to be associated with the infection, although no relationship was established with the course of infection (HCMV disease) (Taniguchi et al., 2013). Eldar-Yedidia et al. (2017) reported a protective effect of TLR2 rs1898830 –GG genotype against HCMV transmission to fetus. A follow-up study investigating the influence of Arg677Trp (rs121917864, 2029 C > T) and Arg753Gln (rs5743708) variants in the TLR2, and Asp299Gly variant in the TLR4 on the risk of CMV infection in infants and adults found that heterozygosity for the TLR2 Arg677Trp was significantly associated with a lower risk of CMV infection in adults but not in infants. The same study also reported TLR4 Asp299Gly association with lower viremia in the adults (Jablonska et al., 2014). In a study of HCMV-infected fetuses and neonates, and controls, Wujcicka et al. (2017a) reported TLR2 2258 G > A SNP (rs5743708) to be associated with increased risk of congenital HCMV infection, but no effect of TLR2 1350 T > C and 2029 C > T variants on HCMV risk was observed. The same group in an independent study evaluated the role of TLR2, TLR4, and TLR9 variants in HCMV infection among pregnant women. Only the TLR9 2848 G > A (rs352140) variant was reported to be associated with HCMV infection risk in pregnant women (Wujcicka et al., 2017b). Increased HCMV infection risk in infants with TLR9 -1486 T > C and TLR9 2848 C > T variants is also reported (Paradowska et al., 2016).

Another important player in the innate immune system is the Mannan-binding lectin (MBL), a pattern recognition molecule and a first line defense antimicrobial factor (Kilpatrick, 2002). Mutations in the promoter region and first exon of *MBL2* were reported to be associated with lower serum MBL concentrations (Madsen et al., 1995). In a Polish study, MBL2 functional gene polymorphisms that influence serum MBL concentrations were examined in prenatal and perinatal CMV infections (Szala et al., 2011). However, no significant influence on susceptibility to prenatal or perinatal HCMV infections was observed (Szala et al., 2011).

HCMV infection during pregnancy can affect the cytokine profile within a HCMV-infected placenta and shift the cytokine expression toward a proinflammatory state with implications for adverse pregnancy outcomes (Hamilton et al., 2012; Scott et al., 2012). As host genetic variants in cytokine-related genes were shown to influence susceptibility to HCMV infection and disease in transplant patients and patients with AIDS, several congenital infection studies also investigated the association of cytokine and cytokine receptor variants on HCMV susceptibility. Kasztelewicz et al. (2017) compared the allelic distribution of 11 candidate SNPs in eight genes (TNF rs1799964 and rs1800629, TNFRSF1A rs4149570, IL-1B rs16944 and rs1143634, IL-10 rs1800896, IL-10RA rs4252279, IL-12B rs3212227, CCL2 rs1024611 and rs13900, CCR5 rs333) between a group of infants (n = 72) with confirmed intrauterine CMV infection and 398 uninfected controls. IL-1B (rs16944) and TNF (rs1799964) variants were significantly associated with intrauterine HCMV infection. Moreover, they identified CCL2 (rs13900) as a genetic risk factor for hearing loss at birth and at 6 months of age (Kasztelewicz et al., 2017). Wujcicka et al. examined the effects of fetal and maternal IL-1A, IL-1B, IL-6, IL-12B, and TNFA gene variants on HCMV infection and disease in neonates and fetuses in two independent Polish cohort studies. In one study, they reported that IL-1A and IL-1B variants increased the risk of congenital HCMV infection in neonates and fetuses, as well as the onset of disease-related symptoms (Wujcicka et al., 2017c). The other study of pregnant women also reported possible effects of IL-1A, IL-1B, and IL-6 on the occurrence and development of HCMV infection in the neonate (Wujcicka et al., 2017d).

Deciphering the contribution of hot genetics to HCMV vertical transmission and related disease outcomes may be the hardest of all HCMV-related disease studies. Firstly, the susceptibility of the pregnant mother to HCMV needs to be considered, where the immune response will be modified due to pregnancy further complicating the interaction between the host and HCMV. Secondly, if the mother cannot clear the infection, and HCMV finds its way to fetus, then the immune response by the infant, which is rather immature and still developing, will be involved with a genetic make-up different than that of the mother. Aforementioned reports should be considered as early attempts of a rather challenging research agenda. High-throughput genetic and immune profiling methods with much larger cohorts are necessary to understand the genetic and non-genetic factors involved in HCMV vertical transmission.

## GWAS of HCMV Infection

Genome-wide association studies (GWASs) have made significant contributions for discovering genetic factors underlying complex phenotypes and diseases. Unlike traditional hypothesis-driven candidate gene studies, where only a few candidate genes are targeted, in GWAS all human genes become potential candidates for the phenotype of interest. Therefore, GWAS approach can discover genes and their variants that may look irrelevant to the phenotype of interest, which in return can lead to discovery of novel biological pathways involved in development of this phenotype.

The first GWAS was conducted to identify genetic polymorphisms associated with the susceptibility to HCMV and strength of anti-HCMV immunoglobulin G (IgG) response to CMV infection (Kuparinen et al., 2012). The study included 1486 anti-CMV IgG seropositive and 648 seronegative individuals genotyped on an Illumina BeadChip containing 670,000 probes. Although no strong genetic components were observed, the study identified 10 new candidate loci that showed suggestive association with anti-CMV IgG titer (**Table 1**). Annotated genes among these loci suggested a possible role for microtubule network in anti-CMV antibody response (Kuparinen et al., 2012). Another GWAS, aiming to localize the loci influencing serological phenotypes to common viral infections, found suggestive evidence of association for modifying IgG antibody response to HCMV (anti-CMV) on chromosome 14 (Rubicz et al., 2015). A retinol metabolism gene, *DHRS4*, near the associated SNP, was proposed to be a candidate for further evaluation. These two studies show that GWAS approach can be productive in HCMV field; however, one also needs to consider the fact that there was no overlap of identified genes between the two GWASs, although the phenotypes were similar. Curiously, none of the candidate innate and adaptive immune genes examined so far were top hits in these GWASs.

### Challenge and Future Directions

Clinical management of CMV infection is particularly challenging due to the arsenal of host immune evasion strategies encoded by its large genome and its complex interactions with its human host. As we show in this review, with a few exceptions, most of the genetic loci identified to date have not been replicated or validated in sufficiently powered cohort studies, suggesting that only a small fraction of variance in host response is likely due to genetic variation. To address the role of host genetic variation in immune response to HCMV and CMV disease, large prospective studies and genome-wide approaches are required to securely identify causal variants involved in immune response and pathophysiological mechanisms leading to CMV disease.

## Author Contributions

ES designed the study, conducted literature research, and wrote and edited the manuscript. PA and CW wrote and edited the manuscript.

## Funding

This project has been funded in whole or in part with federal funds from the National Cancer Institute, National Institutes of Health, under contract HHSN26120080001E. This research was supported in part by the Intramural Research Program of the NIH, National Cancer Institute, Center for Cancer Research. The content of this publication does not necessarily reflect the views or policies of the Department of Health and Human Services, nor does mention of trade names, commercial products, or organizations imply endorsement by the U.S. Government.

The funders had no role in study design, data collection and analysis, decision to publish, or preparation of the manuscript.

## Conflict of Interest Statement

PA and CW are employees of Leidos Biomedical Research, Inc., and declare no competing interest.

The remaining authors declares that the research was conducted in the absence of any commercial or financial relationships that could be construed as a potential conflict of interest.
